# Formation of Aberrant Myotubes by Myoblasts Lacking Myosin VI Is Associated with Alterations in the Cytoskeleton Organization, Myoblast Adhesion and Fusion

**DOI:** 10.3390/cells9071673

**Published:** 2020-07-11

**Authors:** Lilya Lehka, Małgorzata Topolewska, Dominika Wojton, Olena Karatsai, Paloma Alvarez-Suarez, Paweł Pomorski, Maria Jolanta Rędowicz

**Affiliations:** Laboratory of Molecular Basis of Cell Motility, Nencki Institute of Experimental Biology, Polish Academy of Sciences, 3 Pasteur St., 02-093 Warsaw, Poland; l.legka@nencki.edu.pl (L.L.); m.topolewska@nencki.edu.pl (M.T.); d.wojton@nencki.edu.pl (D.W.); o.karatsai@nencki.edu.pl (O.K.); p.alvarez-suarez@nencki.edu.pl (P.A.-S.); p.pomorski@nencki.edu.pl (P.P.)

**Keywords:** adhesion, differentiation, drebrin, FAK, fusion, M-cadherin, myomerger, myomixer, talin, vinculin

## Abstract

We have previously postulated that unconventional myosin VI (MVI) could be involved in myoblast differentiation. Here, we addressed the mechanism(s) of its involvement using primary myoblast culture derived from the hindlimb muscles of Snell’s waltzer mice, the natural MVI knockouts (MVI-KO). We observed that MVI-KO myotubes were formed faster than control heterozygous myoblasts (MVI-WT), with a three-fold increase in the number of myosac-like myotubes with centrally positioned nuclei. There were also changes in the levels of the myogenic transcription factors Pax7, MyoD and myogenin. This was accompanied by changes in the actin cytoskeleton and adhesive structure organization. We observed significant decreases in the levels of proteins involved in focal contact formation, such as talin and focal adhesion kinase (FAK). Interestingly, the levels of proteins involved in intercellular communication, M-cadherin and drebrin, were also affected. Furthermore, time-dependent alterations in the levels of the key proteins for myoblast membrane fusion, myomaker and myomerger, without effect on their cellular localization, were observed. Our data indicate that in the absence of MVI, the mechanisms controlling cytoskeleton organization, as well as myoblast adhesion and fusion, are dysregulated, leading to the formation of aberrant myotubes.

## 1. Introduction

Myosin VI (MVI) is a unique and unconventional myosin, as, unlike all other known myosins, it moves backwards, i.e., towards the minus end of the actin filament. It belongs to a structurally and functionally diverse myosin superfamily formed from over 35 families, members of which are expressed in nearly all eukaryota [1]. Myosins are involved in numerous cellular functions, and the best known is muscle contraction, in which muscle isoforms play a pivotal role. Muscle myosins and so-called non-muscle myosins (NMII), ubiquitously expressed and structurally and functionally resembling muscle isoforms, form class II, the most abundant within the superfamily. Class II myosins, encoded by *MYH* genes, are also defined as conventional, whereas all other myosins are termed unconventional, and are encoded by *MYO* genes. 

Besides muscle isoforms, several other myosins, including two non-muscle myosins (NMIIA and NMIIB) and several unconventional myosins, such as myosin I isoforms, myosin VA, and myosin XVIIIA and XVIIIB, were shown to be expressed and to function in the muscle [2,3,4,5,6,7]. Furthermore, we have shown that MVI is expressed in skeletal muscles, where it seems to be involved in the functions of the sarcoplasmic reticulum (SR) and neuromuscular junction, and possibly in gene transcription [8,9]. Interestingly, a point mutation (H246R) within *MYO6* has been associated with cardiac hypertrophy, suggesting the important role of this molecular motor in striated muscles [10]. It was later shown that in cardiac muscle, MVI is located in the SR and intercalated discs [9,11,12]. MVI is also present in myogenic cells, where it is postulated to play a role in myoblast differentiation [13]. 

MVI is encoded by a single gene (*MYO6*), ubiquitously expressed in all metazoans, and exhibits a domain organization pattern similar to all other myosins [14]. Its 140-kDa heavy chain (MW ~140 kDa) contains (i) an N-terminal motor domain (with the actin- and ATP-binding sites), (ii) a neck, which binds two calmodulin molecules, and (iii) a tail domain, with the C-terminal part forming a globular domain involved in cargo binding. There is a 53-amino acid insert within the neck domain that is responsible for MVI’s unique ability to move backwards on actin filaments [15]. Further, four splice variants of MVI can be formed in mammalian cells due to the presence of two inserts (small and large) within the tail domain. It has been postulated that the inserts determine MVI subcellular localization, and thus function [16,17]. We showed that in undifferentiated myoblasts, all four isoforms were expressed, and in myotubes and adult skeletal muscle, the forms with a large insert are missing [8,13].

Mutations within *MYO6* lead to hearing impairment in mammals, due to the disintegration of the inner ear hair cell stereocilia [18]. Snell’s waltzer mice (*SV*) lacking functional MVI also have several other defects, such as cardiac hypertrophy, as well as brain, kidney and spermiogenesis dysfunctions [11,12,19,20,21,22]. MVI is involved in endocytosis and the intracellular transport of vesicles and organelles, cell migration, the maintenance of Golgi apparatus, actin cytoskeleton organization, and possibly in gene transcription [23,24,25,26,27,28]. MVI fulfills its functions through interaction with actin (binding to the N-terminal motor domain in the ATP-dependent manner), and its partner proteins (binding to the C-terminal cargo domain through a positively charged RRL region and a hydrophobic WWY region) [29]. Additionally, a positively charged cluster of the MVI C-terminal globular tail was shown to bind to PIP2-containing liposomes, possibly aiding in the binding partners recognition [30]. MVI can act in vitro as a monomer or a dimer, but it has been shown that it must dimerize and deploy its unusual lever arm in order to perform its cellular functions [31]. 

Numerous tissue/cell-specific MVI binding partners have been identified in mammals; among them are the adaptor proteins, enzymes and proteins involved in the regulation of cytoskeleton dynamics [29,32]. In rat skeletal muscle, MVI seems to interact with target of myb1 homolog isoform 1 (TOM1), a protein involved in intracellular transport and autophagy, as well as with fragile X mental retardation protein involved in mRNA transport (FMRP) and hnRNP proteins, heterogeneous ribonucleoproteins involved in RNA transport and maturation [8]. In murine C2C12 myoblasts, MVI interacts with numerous proteins involved in various cell processes, and the interaction pattern depends on the myoblast differentiation state. Only nine partners interact with MVI during the entire differentiation process [33]. Among them is talin, a protein involved in the focal adhesive structure formation, and its localization and level are altered in MVI-depleted C2C12 cells. 

Myoblast fusion into myotubes is one of the crucial steps of myogenesis. The fusion is preceded by the specification of a myogenic lineage (mesodermal progenitors) that is capable of differentiation into myoblasts; in adult muscles, there are satellite cells that reside underneath the basal lamina of muscle fiber, and which are activated in response to various stimuli. The main cellular events taking place during myoblast fusion include cell adhesion and membrane fusion [34]. Thus, it is a complex process that is tightly regulated by a network of signaling pathways, with a plethora of genes and their products being involved, and with many of them implicated in cell migration and adhesion [34,35]. Myoblast differentiation, including the fusion, is under the control of myogenic regulatory factors (MRF), such as myogenin, MyoD and MRF4 [36]. 

Since our earlier observations indicate that MVI could play a role in myoblast fusion [13], we attempted to identify the mechanisms of its involvement in the fusion process. Our data on the primary myoblast culture derived from the hindlimb muscles of Snell’s waltzer mice show alterations in myoblast fusion, resulting in the formation of aberrant myotubes, accompanied by dysregulation of the level of proteins involved in the cytoskeleton organization, adhesion, and cell–cell communication, as well as membrane fusion. 

## 2. Materials and Methods

### 2.1. Animals 

Three-month-old male Snell’s waltzer mice (C57BL/6 background) were used in the study. These mice do not synthesize MVI due to a 130-bp deletion which results in the introduction of a stop codon in the neck region of MVI and serves as a natural MVI knockout. Each experiment was performed at least three times using a pair of control (heterozygous: sv/+, WT) and mutant (sv/sv, KO) males from one litter. The mice, a gift from Dr. Folma Buss from Cambridge Institute for Medical Research, University of Cambridge, UK, were bred and housed under pathogen-free conditions in the animal facility of the Nencki Institute. Animal housing and sacrifice procedures were performed in compliance with the European Communities Council directives adopted by the Polish Parliament (Act of January 15, 2015 on the use of animals in scientific investigations), and got approval from the Director of the Nencki Institute of Experimental Biology with the approval number: NR 155/2015/IBD. 

### 2.2. Myoblast Isolation 

The myoblast-enriched cultures (referred to herein as primary myoblast cultures) were obtained from Snell’s waltzer hindlimb muscles (gastrocnemius, tibialis anterior, soleus and extensor digitorum longus muscles). The muscles were dissected from three-month old mice and dissociated using Skeletal Muscle Dissociation Kit (MACS Miltenyi Biotec 130-098-305) according to the manufacturer’s instructions. After that, plating media containing DMEM (Gibco 21885025), 10% horse serum (HS; Gibco 26050088), 0.5% chicken embryonic extract (CEE; MP Biomedicals 092850145) and 1% penicillin/streptomycin (Thermo Fisher 15140122) was added to the cell pellet. The isolated cell mixture was first plated into a regular TC-treated dish (Sarstedt 83.3902) overnight at 37 °C and with 5% CO_2_ to eliminate fibroblasts. Then, the supernatant containing myoblasts that, unlike fibroblasts and adipocytes, did not attach to the surface was collected and centrifugated at 700*g* for 20 min. The obtained pellet was resuspended in a differentiation medium containing DMEM, 10% HS, 20% fetal bovine serum (FBS; Gibco 10500064) and 0.5% CEE and transferred into 12-well plates or 6-cm Petri dishes (dependent on the aim of an experiment) coated with 5% Matrigel (Corning 356230). 

### 2.3. Microscopy and Imaging

The microphotographs of differentiating myoblasts were taken on indicated days using a Nikon Eclipse Ti-U inverted fluorescence microscope and a Nikon Digital Sight DS-U3 camera (Nikon Corporation, Shinagawa, Tokyo, Japan). Archiving was performed in the NIS-Elements Basic Research program dedicated to this microscope. For the imaging of immunofluorescence cell samples on glass slides, a LSM780 confocal microscope equipped with 10×/0.30 EC Plan-Neofluar, 40×/1.4 and 63×/1.4 Oil Plan Apochromat DIC objectives was used. The images were processed using ZEN Black 3.0 SR or Zen Blue 3.1 (Carl Zeiss Microscopy GmbH, Jena, Germany) software,. Confocal image series were enhanced by three-dimensional (3D) deconvolution using Huygens Professional 14.10 software (Scientific Volume Imaging, Hilversum, Netherlands,) by applying a classic maximum-likelihood estimation algorithm and an automatically-generated point-spread function to optimize z-axis images. Then rotations and z-axis resampling were performed using Fiji distribution of ImageJ software [37,38].

**Figure 1:**
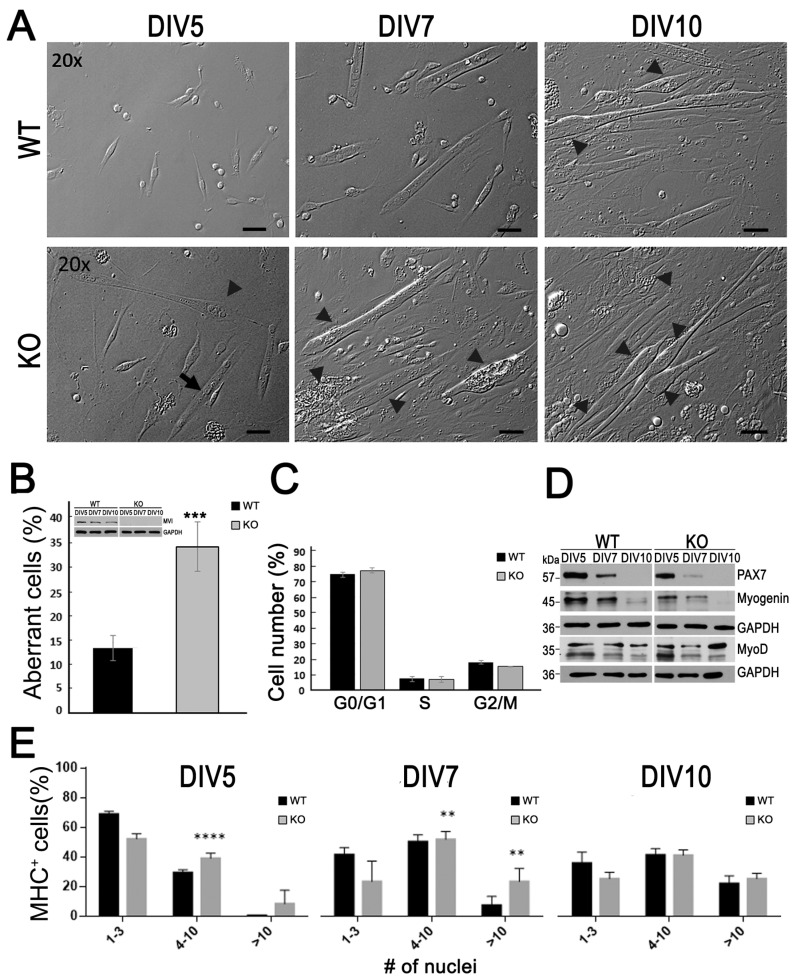
Effect of myosin VI (MVI) loss on myoblast differentiation. (**A**) Micrographs of heterozygous (WT) and MVI knockout (KO) myoblasts cultured for up to 10 days (DIV5–DIV10). The arrow points to a nascent myotube; arrowheads point to aberrant myotubes; Bars, 20 µm. (**B**) Quantification of aberrant myotubes at DIV10. Inset, immunoblotting for MVI in WT and KO cells. (**C**) Cell cycle analysis of WT and KO cells at DIV7. (**D**) Analysis of the levels of myogenic transcription factors during WT and KO myoblast differentiation. This is a representative blot from three independent experiments. (**E**) Analysis of fusion efficiency. In B, C and E, three independent experiments were performed. In B and D, GAPDH served as in internal loading control. **, *p* ≤ 0.01; ***, *p* ≤ 0.001; ****, *p* ≤ 0.0001. Other details are in Section 2.

To estimate the myoblast fusion efficiency as well as myotube width and length in the primary myoblast culture during in vitro differentiation, the myoblasts were stained for DAPI and fast myosin heavy chain (MHC), and stained myotubes were grouped into three subgroups based on the number of nuclei within each MHC^+^ cell; 1–3, 4–10 and more than 10 nuclei (Figure 1). The fraction of each subgroup was calculated for WT and KO myotubes with respect to the total number of myotubes within each image taken by Nikon Eclipse Ti-U microscope equipped with 20×/0.45 HMC ELWD Plan Fluor objective, using ImageJ software. At least 15 separate view fields from two replicates for every sample were analyzed. 

A fraction of aberrant cells (in%) was calculated as the number of cells with a myosacs-like morphology with respect to the total amount of myotubes within each of set images taken by a Nikon Eclipse Ti-U microscope of the day-10 culture. At least 50 microphotographs, and a total number of 250 myotubes for each WT and KO samples, were analyzed. 

### 2.4. Immunoblotting

At indicated days cells were lysed in an ice-cold RIPA buffer containing 50 mM Tris-HCl pH 7.5, 150 mM NaCl, 0.5% sodium deoxycholate, 0.1% SDS, 1% Nonidet P-40, 50 mM sodium fluoride and 1 mM PMSF supplemented with protease (Roche 04693116001) and phosphatase (Roche 04906837001) inhibitors, boiled in SDS loading buffer. Cell lysates (10–30 µg of protein per well) were separated using 10% or 12% polyacrylamide SDS-gels and then transferred to a nitrocellulose membrane (GE Healthcare 10600002). Western blot was performed as in [8], and the bands were detected by the enhanced chemiluminescence technique (ECL, Millipore, WBKLS0500) based on the activity of horse radish peroxidase conjugated with secondary antibodies. 

### 2.5. Antibodies and Fluorescent Markers

The antibodies used were as follows: mouse monoclonal antibody to Pax7 (Santa Cruz sc-81648, 1:500); mouse monoclonal to MyoD (Abcam ab16148, 1:1000); mouse monoclonal to myogenin (Santa Cruz sc-52903, 1:500); goat polyclonal to talin (Santa Cruz sc-7534, WB 1:200, IF 1:50); mouse monoclonal to vinculin (Sigma-Aldrich V4505, 1:2000); rabbit polyclonal to focal adhesion kinase (FAK) (Cell Signaling #3285, 1:1000); rabbit monoclonal to phosphor-FAK (Tyr397) (Cell Signaling #8556, 1:1000); mouse monoclonal to M-cadherin (BD Bioscience 611100, 1:500); sheep polyclonal to myomixer/myomerger (ESGP) (Novus Biologicals AF4580, WB 1:125, IF 1:50); rabbit polyclonal to myomaker (TMEM8C) (Novus Biologicals NBP2-34175, WB 1:125, IF 1:50); mouse monoclonal to GAPDH (Millipore MAB374, 1:15,000); mouse monoclonal to fast myosin skeletal heavy chain (Abcam ab51263, IF 1:50); mouse monoclonal to slow skeletal myosin heavy chain (Abcam ab11083, 1:1000); mouse monoclonal to sarcomeric α-actinin (Abcam ab9465, 1:1000); mouse monoclonal to desmin (BD Biosciences 550626, 1:1000); mouse monoclonal to γ-actin (Sigma-Aldrich A8481, 1:1000); mouse monoclonal to β-actin (Sigma-Aldrich A5441, 1:1000); rabbit monoclonal to dysferlin (Abcam ab124684, 1:500); mouse monoclonal to drebrin (GeneTex GTX12350, 1:250), goat anti-mouse IgG Antibody, HRP conjugate (Millipore AP308P, 1:10,000); goat anti-rabbit IgG antibody, HRP conjugate (Millipore AP307P, 1:10,000); donkey anti-goat IgG antibody, HRP conjugate (Santa Cruz sc-2020, 1:10,000). For the fluorescent staining of actin filaments in myoblasts, Alexa Fluor 488-conjugated phalloidin (Invitrogen A12379, 1:40) and Alexa Fluor 546 conjugated phalloidin (Invitrogen A22283, 1:40) were used. Alexa Fluor 488- and Alexa Fluor 555-conjugated secondary antibodies were used to detect primary antobodies (Invitrogen A11008 and Invitrogen A21432, 1:1000, respectively). 

For immunofluorescence stainings, cells plated on glass coverslips were washed three times in PBS and fixed with 4% PFA in PBS for 15 min. After that, 50 mM ammonium chloride was added followed by 30 min incubation. Then, cells were quickly washed twice with PBS, with subsequent permeabilization with 0.2% Triton X-100 in PBS for 10 min. After three washes with PBS, cells were blocked with 3% freshly made heat-inactivated normal horse or 3% goat serum, or 2% BSA in PBS with 0.02% Triton X-100 for 1–2 h, and incubated with primary antibodies overnight at 4 °C. The next day, after three washes with PBS, cells were incubated with Alexa Fluor488- or Alexa Fluor 546-conjugated secondary antibodies for 1–2 h at room temperature. After three washes with PBS, cells on the coverslips were mounted with Vectashield antifade mounting medium with DAPI (Vector Labs H-1200), and stored at 4 °C for further analysis. 

### 2.6. Cell Cycle Analysis

Myoblasts were harvested at day 7, washed twice in ice-cold PBS and then fixed by resuspension in ice-cold 70% ethanol and incubated overnight at 4 °C. Cells were then washed in PBS, centrifuged at 300× *g* and resuspended in PBS. Then RNase and propidium iodide were added to final concentrations of 5 and 50 μg/mL, respectively. After 30-min incubation, propidium iodide fluorescence of 10,000 cells per sample was measured on Guava^®^ easyCyte^™^ 8HT Benchtop Flow Cytometer (Merck Millipore, Burlington, MA, USA) The obtained data were processed using InCyte 2.7 software (Merck Millipore, Burlington, MA, USA).

### 2.7. Statistical Analysis

Each value reported represents the mean ±S.D. of more than two independent replicates for each experiment. Quantitative data were analyzed by unpaired two-tailed Student’s t-test. All statistical analyses were performed using GraphPad Prism 8.4.3 Software (GraphPad San Diego, CA, USA). *p* < 0.05 was considered statistically significant. *p* < 0.05 was marked with *, *p* ≤ 0.01 was marked as **, *p* ≤ 0.001 was marked as *** and *p* ≤ 0.0001 as ****.

## 3. Results

### 3.1. Aberrant Myotube Formation by Myoblasts Lacking MVI

To examine the role of MVI in myotube formation, a primary myoblast culture was established from the hindlimb muscle obtained from three-month-old Snell’s waltzer male mice, which serve as a natural MVI knockout (KO) [14]. All the experiments were performed with respect to a myoblast primary culture derived from heterozygous male animals from the same litter; these myoblasts were considered as wildtypes (WT). Isolated WT and KO myoblasts were cultured for up to 10 days (see Figure 1).

As shown in Figure 1A, the course of myotube formation and the morphology of myotubes were significantly altered in KO myoblasts. While in 5-day WT culture the predominant forms were oval and elongated myoblasts, in KO culture, both the nascent and abnormal myotubes with centrally situated nuclei, resembling myosac-like structures [39], were already present (Figure 1A, arrow and arrowhead, respectively). These abnormally thick myotubes with myosac morphology were even more visible in the 7- and 10-day KO cell cultures (Figure 1A, arrowheads). Their diameters at the widest point could reach 30 µm (Figure S1A). Quantification of the myotube images revealed that at day 10, about 14% and 35% abnormal myotubes were formed in the WT and KO cultures, respectively (Figure 1B). 

To check whether the observed difference in myoblast differentiation could result from alterations in the cell cycle, we analyzed the cycle progression by means of flow cytometry, with the use of propidium iodide. As shown in Figure 1C, there was not a significant difference in the stages of the cycle at day 7, when the aforementioned differences were already pronounced. 

To understand the mechanisms of the observed difference in myotube formation, we examined the levels of myogenic transcription factors in both the culture types up to day 10 (Figure 1D and Figure S2). The level of Pax7, serving as the myoblast marker, was lower in the 5-day and 7-day KO myoblasts, and the protein was not detectable at day 10 in either cell type. The level of myogenin, the myocyte marker, was lower in KO cells at all the examined time points. However, the level of MyoD, also a myocyte marker, was similar at days 5 and 7, and higher in KO myoblasts at day 10. These data, showing changes in the expression pattern of the examined transcription factors, indicate that the process of myoblast differentiation could be affected by the loss of MVI.

To further quantify the effect of the loss of MVI on myoblast differentiation, an analysis of the number of nuclei in the myotubes stained for fast skeletal muscle myosin heavy chain was next performed (Figure 1E and Figure S1B). As shown in Figure 1E, at day 5, there was already more KO cells containing higher numbers of the nuclei than there was WT cells. The difference was even more pronounced at day 7, but started to be less significant beyond that day, with no statistical significance at day 10 (Figure 1D and Figure S1B). 

**Figure 2:**
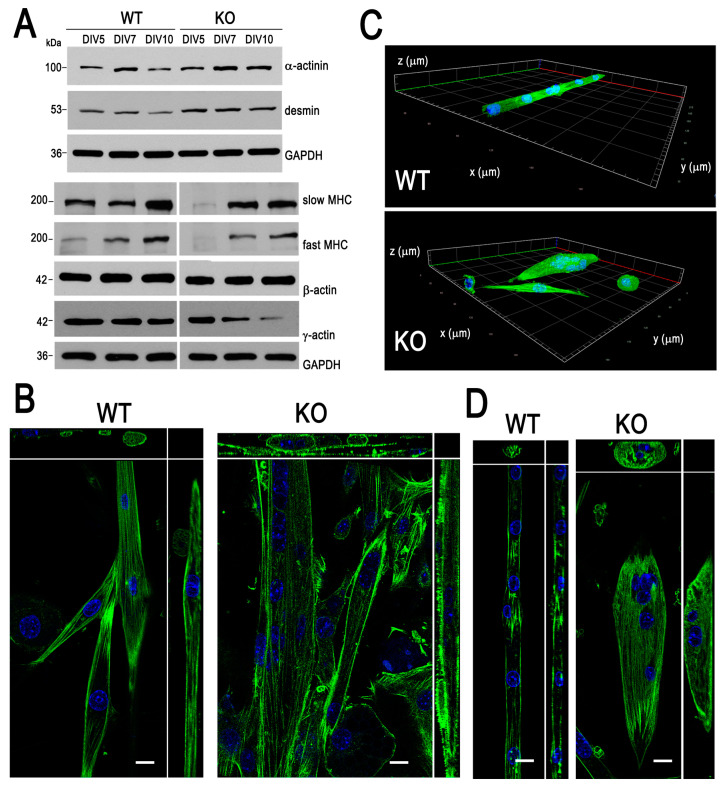
Effects of the lack of myosin MVI (MVI) on cytoskeleton organization. (**A**) Analysis of the levels of cytoplasmic and contractile proteins during differentiation of heterozygous (WT) and MVI knockout (KO) myoblasts. This is a representative blot from three independent experiments. GAPDH served as in internal loading control. (**B**,**D**) present 0.3-µm thick confocal images of the cell centers of WT and KO cells, stained with Alexa Fluor 488-conjugated phalloidin (green) and DAPI (blue) at DIV7 and DIV10, respectively. The images are presented with x–z and y–z projections. Bars, 10 µm. (**C**) The 3D presentation of the WT and KO myotubes at DIV7 stained for actin (green) and nuclei (blue). Other details are in Section 2.

We have also analyzed the levels of cytoskeletal proteins, such as α-actinin (actin-filament crosslinker and Z-disc structural protein), desmin (intermediate filament protein), slow and fast skeletal muscle myosin heavy chains, and both types of cytoskeletal actins, β and γ (Figure 2A and Figure S3A). The Western blot analysis revealed that the levels of α-actinin, and in particular of desmin, were increased. The levels of skeletal myosin heavy chains, and γ-actin, which is more predominant in the muscle cytoskeleton [40], were decreased in KO cells with respect to their WT counterparts. 

The staining of the 7-day myoblasts for microfilaments with Alexa Fluor 488-conjugated phalloidin and nuclei with DAPI further confirmed the differences in the course of myotube formation (Figure 2B–D). As shown in Figure 2B, the fusion seemed to proceed in a less organized manner, and KO myoblasts more readily overlapped each other. The formed structures could be several times thicker than the normal WT myotubes (see Figure 2C,D and Figure S3B). The 3D analysis revealed that the aberrant myotubes could have a spindle-like shape, with the nuclei grouped at the center (Figure 2C, and Figure S3B). 

### 3.2. Cell Adhesion Is Impaired in Myoblasts Lacking MVI

Cell adhesion is one of the crucial steps of myoblast fusion. We and other groups demonstrated that MVI is involved in cell adhesion to the surface, as well as in cell–cell interactions [13,41,42]. Moreover, we showed that talin, one of the major constituents of adhesive structures, is the MVI interaction partner [13]. Therefore, our next step was the evaluation of myoblast adhesion in the examined cells. 

**Figure 3:**
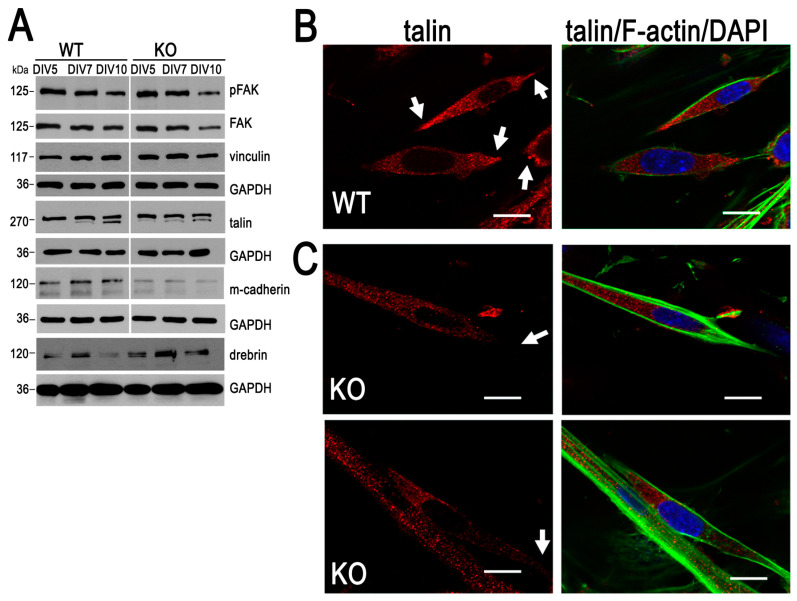
Loss of myosin VI (MVI) affects myoblast adhesion. (**A**) Analysis of the level of proteins involved in cell adhesion and cell–cell contacts during differentiation of heterozygous (WT) and MVI knockout (KO) myoblasts. This is a representative blot from three independent experiments. GAPDH served as in internal loading control. (**B**,**C**) WT and KO myoblasts, respectively, were stained at DIV7 with the antibody against talin (red), Alexa Fluor 488-conjugated phalloidin (green) and DAPI (blue). These are 0.3-µm thick confocal images of the cell regions next to the glass surface. Arrows point to myoblast edges. Bars, 10 µm. Other details are in Section 2.

As shown in Figure 3A and Figure S4, there was not an evident difference in the level of vinculin, a constituent of adhesive contacts. Analysis of talin content revealed a drop in its expression in the 7- and 10-day KO cells (Figure 3A and Figure S4). The difference was also observed for focal adhesion kinase (FAK), the major regulator of adhesive structure organization, and its phosphorylated (active) form (pFAK). Their levels decreased during differentiation in both cell types, but the decrease was more evident in 10-day KO cells (Figure 3A and Figure S4). Intriguingly, the amount of M-cadherin, a protein involved in the myoblast–myoblast contact formation, was substantially lower at the examined time points of KO cells’ differentiation, with respect to their WT counterparts (Figure 3A and Figure S4). Since drebrin was shown to be involved in myoblast adhesion and cell–cell contact stabilization [43,44], we also evaluated the level of this protein in the examined cells. As shown in Figure 3A and Figure S4, the amount of drebrin was elevated in the KO myoblast throughout the differentiation. 

To visualize the adhesive contacts, at day 7 the cells were immunostained for talin (Figure 3B,C). We examined its localization in elongated myoblasts, known to be prone for fusion, and specifically at the edges (marked with arrows) that are enriched in the adhesive structures [45]. As shown in Figure 3C, the talin-associated fluorescence was substantially reduced at the edges of KO myoblasts with respect to the WT cells (Figure 3B). These data indicate that alterations in KO myoblast adhesion could contribute to the formation of aberrant myotubes. 

### 3.3. Expression Pattern of Myomaker and Myomerger Is Altered During Differentiation of Myoblasts Lacking MVI

**Figure 4:**
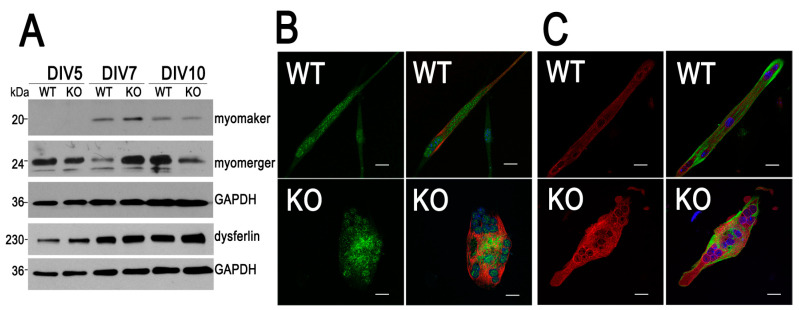
Loss of myosin MVI (MVI) affects expression of proteins involved in myoblast membrane fusion. (**A**) Analysis of the levels of proteins involved in myoblast membrane fusion during differentiation of heterozygous (WT) and MVI knockout (KO) myoblasts. This is a representative blot from three independent experiments. GAPDH served as in internal loading control. (**B**) WT and KO myoblasts were stained at DIV7 with the antibody against myomaker (green), Alexa Fluor 546-conjugated phalloidin (red) and DAPI (blue). (**C**) WT and KO myoblasts were stained at day 10 with the antibody against myomerger (red), Alexa Fluor 488-conjugated phalloidin (green) and DAPI (blue). These are 0.3-µm thick confocal images of the cell centers. Bars, 10 µm. Other details are in Section 2.

Myomaker and myomerger/myomixer/minion (referred to herein as myomerger) are the key proteins involved in myoblast membrane fusion [46]. Moreover, the phenotype observed for KO myotubes resembles that of C2C12 myotubes overexpressing myomaker [47]. In order to check whether membrane fusion could also be impaired in KO myoblasts, we examined the levels of both proteins during myotube formation. As presented in Figure 4A and Figure S5, the levels of these proteins in both types of cells changed during this process. On day 5, the amount of myomaker in both cell types was below the detection level. On day 7, it was higher in KO myoblasts, and on day 10 it decreased to a level similar to that found in WT myoblasts at this time-point. There was also a time-dependent difference in the level of myomerger (Figure 4A and Figure S5). While its amount was similar at day 5 in both examined samples, it was significantly higher in KO cells at day 7, but lower at day 10, whereat the substantial increase of myomerger in WT cells was observed. 

Since there is a direct link of myomerger with dysferlin, a member of the ferlin family involved in membrane repair [48,49], we assessed its level in both the WT and KO samples. As presented in Figure 4A and Figure S5, the amount of dysferlin during myoblast differentiation seems to be slightly higher in KO cells.

We then examined the localization of myomaker and myomerger in WT and KO myoblasts at day 7, when the highest difference in their expression was observed (Figure 4B,C). We did not observe any substantial difference between the localizations of both proteins in WT and KO cells. Myomaker was mainly present in the cytoplasm, the perinuclear region and within the nucleus (Figure 4B), and myomerger localized to the cytoplasm and perinuclear region (Figure 4C). 

The data gathered herein indicate that a lack of MVI results in the formation of aberrant myotubes, and affects the expression of proteins involved in myoblast differentiation, cytoskeleton organization, cell adhesion and cell contacts, as well as myoblast membrane fusion.

## 4. Discussion

In this study, we showed that the loss of MVI leads to the formation of aberrant myotubes, and our data indicate that alterations in myoblast cytoskeleton organization, adhesion and membrane fusion could contribute to the mechanisms behind the observed phenotype. 

A substantial pool of KO myoblasts differentiated into aberrant myotubes with a myosac-like morphology, with misaligned, centrally positioned nuclei. A myosac phenotype was first described in the 1980s for myotubes cultured in the presence of phorbol esters [39]. Several proteins were found to be associated with this phenotype, including four and a half LIM protein 1 (FHL1/SLIM1) and myomaker [47,50]. Noticeably, both proteins are important in muscle functions, as FHL1/SLIM1 is involved in sarcomere assembly, and myomaker is crucial for myoblast membrane fusion during myotube formation. Similar aberrations in myotube morphology were visible after transfection of MVI-depleted C2C12 myoblasts with a construct encoding the H246R MVI mutant, associated with hypertrophic cardiomyopathy in humans [10,13].

Mislocalization of the nuclei indicates a possible dysfunction of the nuclear alignment on a central axis, and a spreading as the myotube elongates; these processes are strictly dependent on the proper organization of the cytoskeleton and forces generated by the actomyosin-based contractility [51]. Numerous studies, including ours, showed that either a depletion or lack of MVI affects the organization of the actin cytoskeleton in numerous cell types, including myoblasts [13,52]. Furthermore, MVI was shown to be involved in the differentiation of several tissues, including spermiogenesis [21,22] and neuritogenesis [53,54]. All the authors postulated mechanisms associated with the regulation of the cytoskeleton organization [21,22,53,54]. Here, we observed changes in the levels of proteins important for cytoskeleton organization and contractile functions, including α-actinin, desmin and γ-actin, as well as fast and slow myosin heavy chains. These differences might contribute to impairment of the generation of the contractile force required, among other things, for nuclei translocation during myotube maturation. 

Myoblast differentiation, both in vitro and in vivo, is under the control of several transcription factors, including Pax7, myogenin and MyoD, with the latter ones being muscle-specific [36]. Pax7 is involved in the regulation of muscle precursor cells proliferation (muscle cell lineage specification), and its expression precedes the synthesis of MyoD and myogenin [55]. We observed that the levels of Pax7 and myogenin were slightly lower in KO myoblasts, while the level of MyoD was higher, in particular at day 10. These observations suggest changes in the expression pattern of muscle-specific genes that are under the control of the examined factors. However, they were not associated with the cell cycle progression, indicating that proliferation was not affected. It is noteworthy that this is in agreement with our earlier report on the C2C12 cell line with MVI knockdown [13]. The mechanisms of the observed changes in the expression levels of the myogenic factors remain to be unveiled, but one possibility is that MVI could participate in transcription, as was shown for several cancer cell lines, including neurosecretory rat pheochromocytoma PC12 cells [24,28]. However, this is rather unlikely as, in contrast to numerous cancer cells, MVI is not present in myoblast nuclei [13]. Furthermore, it cannot be neglected that mislocalization of the nuclei could affect the course of transcription, as several reports link defects in nuclear positioning with muscle dysfunction [51]. 

Earlier reports indicated that MVI is involved in cell adhesion and cell–cell contact formation, through interaction with vinculin, cadherins and connexin-43 [12,41,42,56,57]. We also showed that talin is a potential MVI binding partner, and its absence from the cell periphery and the formation of smaller adhesive structures was observed in MVI-depleted myoblasts [13]. Interestingly, the interaction of talin with *Dictyostelium discoideum* myosin VII, another unconventional myosin, was reported to play an important role in adhesion complex dynamics [58,59]. Furthermore, two non-muscle myosin II isoforms, NMIIA and NMIIB, are involved in myoblast elongation and adhesion, and thus fusion [60]. In KO myotubes, the amount of talin was reduced, as was its presence in the myoblast edges. This difference could result from impairment of talin targeting to, and/or its maintenance at, the cell periphery. It was associated with a decrease in the level (and activity) of FAK, the key kinase regulating the assembly of adhesive structures, also in myogenic cells, and in particular of M-cadherin, a membrane protein regulating cell–cell contact formation and recognition [61,62]. These observations indicate that in the absence of MVI, cell adhesion and intercellular contacts are impaired. However, surprisingly, the amount of drebrin, an actin-binding MyoD-dependent protein, was higher in KO myoblasts throughout the differentiation. Drebrin, present at actin-rich cellular projections and at regions of cell–cell contact, was shown to be involved in myoblast differentiation, as its depletion severely impaired myotube formation [63]. It is postulated to be involved in myoblast adhesion and cell–cell contact stabilization [43,44]. Thus, it is plausible that in KO myoblasts, a higher expression of drebrin could, to a certain extent, compensate for the decreased amount of M-cadherin. 

We observed for the very first time a link between MVI and the proteins specifically involved in the myoblast membrane fusion, myomaker and myomerger. The expression of both proteins is controlled by MyoD and myogenin, and follows the expression of these factors and their content changes during muscle development and repair [46]. Interestingly, aberrant myotubes formed by KO myoblasts resembled the ones formed by myoblasts overexpressing myomaker, suggesting a link between MVI and myomaker [47]. We observed that the expression pattern of myomaker and myomerger, a functional partner of myomaker in the membrane fusion, was affected by the loss of MVI. This could be connected with MyoD expression, which is relatively high in differentiating KO cells. Both proteins are known for their cytoplasmic and perinuclear localization, and this was not affected by the loss of MVI, indicating that MVI is not involved in their cellular targeting. Of note, we performed a pull down assay with the MVI cargo domain as bait, to assess whether myomaker and/or myomerger could be MVI binding partners, but we did not find any of them in the eluates. We also assessed the level of dysferlin, a protein involved in membrane repair, which is postulated to have a direct link with myomerger [48,49]. In our study, we did not find a correlation between the expression patterns of myomerger and dysferlin. However, a potential link between dysferlin and MVI could exist, as the expression of dysferlin seems to be slightly increased in the KO cells. 

## 5. Conclusions

Our data demonstrate that MVI is involved in myoblast differentiation since its loss leads to the formation of aberrant myotubes, with the myosac morphology characterized by a spindle-like shape with centrally positioned nuclei. This is accompanied by the changes in the expression patterns of transcription factors involved in myoblast differentiation (Pax7, MyoD and myogenin), as well as proteins involved in the cytoskeleton organization (α-actinin, desmin, myosin heavy chains and γ-actin), cell adhesion and cell–cell interaction (talin, FAK kinase and its active form, M-cadherin and drebrin), and in myoblast membrane fusion (myomaker and myomerger). Moreover, we observed the MVI-dependent upregulation of the fusion events, and a decrease of talin-containing adhesive structures. We propose that MVI plays a role in myoblast differentiation through its involvement in cytoskeleton organization, cell adhesion and intercellular communication, as well as in myoblast membrane fusion.

## Supplementary Materials

The following are available online at https://www.mdpi.com/2073-4409/9/7/1673/s1, Figure S1: Assessment of primary mouse myoblasts during differentiation, Figure S2: Densitometric analysis of the levels of Pax7, myogenin and MyoD myoblast during differentiation, Figure S3: Analysis of cytoskeletal proteins, Figure S4: Densitometric analysis of the levels of proteins involved in cell adhesion and cell–cell contacts and Figure S5: Densitometric analysis of the levels of proteins involved in myoblast membrane fusion and repair.
