# Supplementary material for: Formation of Aberrant Myotubes by Myoblasts Lacking Myosin VI Is Associated with Alterations in the Cytoskeleton Organization, Myoblast Adhesion and Fusion
Source: Cells. 2020 Jul 11;9(7):1673. doi: 10.3390/cells9071673 (PMC7408620; doi:10.3390/cells9071673)
Supplement: Supplementary file 1 [file cells-09-01673-s001.zip › Supplementary Figure 1.pdf]

Lehka et al. Supplementary Figure 1

Figure S1. Assessment of primary mouse myoblasts during differentiation.

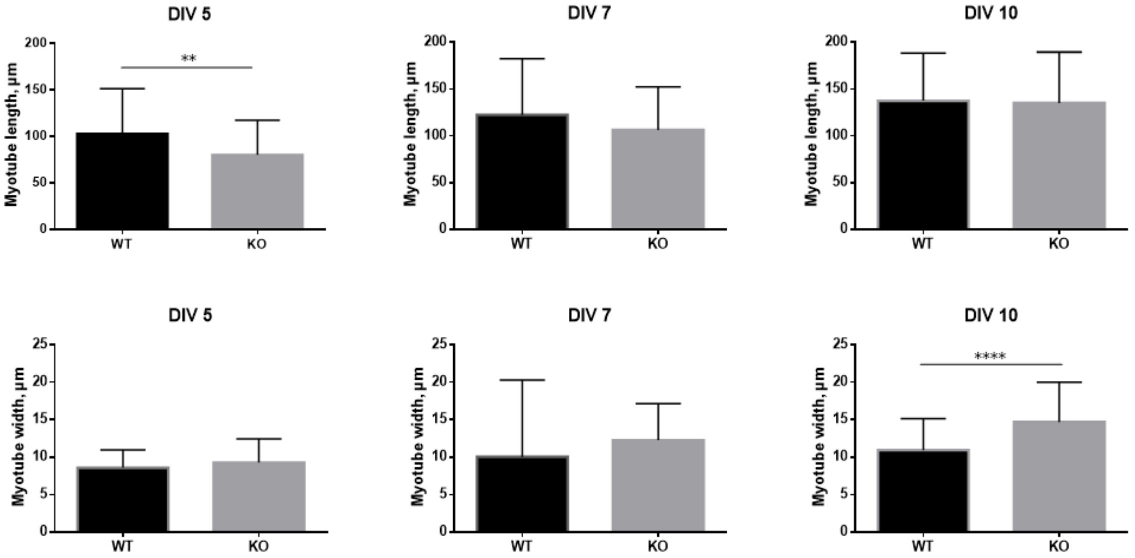

Figure S1A. Quantitative analysis of myotube length and width. Myoblasts derived from hindlimb muscles of 3 month old heterozygous (WT) and *Snell's waltzer* (KO) mice were cultured up to 10 days, stained at indicated DIVs for fast myosin heavy chain (MHC) and nuclei (DAPI). For estimation of the mean values of the myotube width (in the widest part of myotube) and length, the MHC^+ myotubes with more than 3 nuclei were analyzed in 15 view fields for each of two replicates using ImageJ software. The data are presented as mean ±SD versus control (WT cells); t-test, ***p*<0.01,*****p*<0.0001.

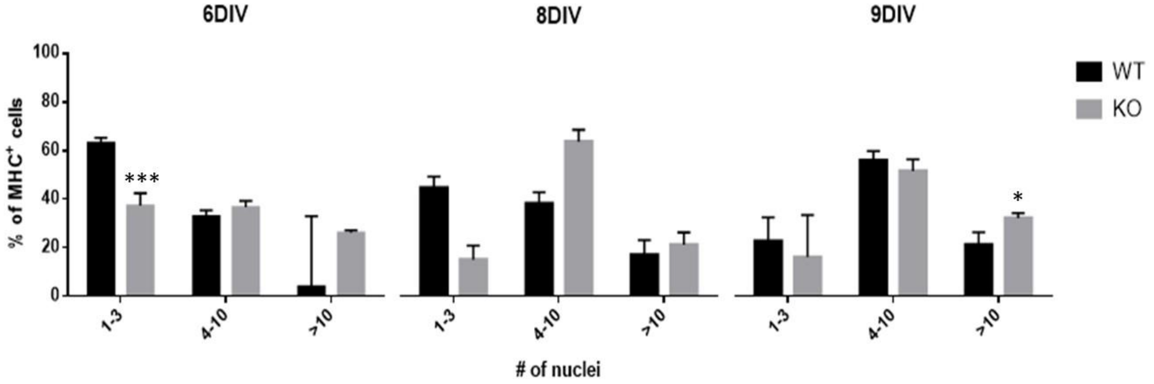

Figure S1B. Myoblasts were derived from and stained as in Figure S1A, and analysis of the number of the nuclei in MHC^+ cells was performed with ImageJ software. Analysis of the number of nuclei in DIV5, DIV7 and DIV10 cells is presented in Figure 1E of the manuscript. Details are described in the Materials and Methods section. The data are presented as mean ±SD versus control (WT cells); t-test, **p*<0.05, ****p*<0.001.
