# Supplementary material for: Formation of Aberrant Myotubes by Myoblasts Lacking Myosin VI Is Associated with Alterations in the Cytoskeleton Organization, Myoblast Adhesion and Fusion
Source: Cells. 2020 Jul 11;9(7):1673. doi: 10.3390/cells9071673 (PMC7408620; doi:10.3390/cells9071673)
Supplement: Supplementary file 1 [file cells-09-01673-s001.zip › Supplementary Figure 2.pdf]

Lehka et al. Supplementary Figure 2

Figure S2. Densitometric analysis of the levels of Pax7, myogenin and MyoD during myoblast differentiation.

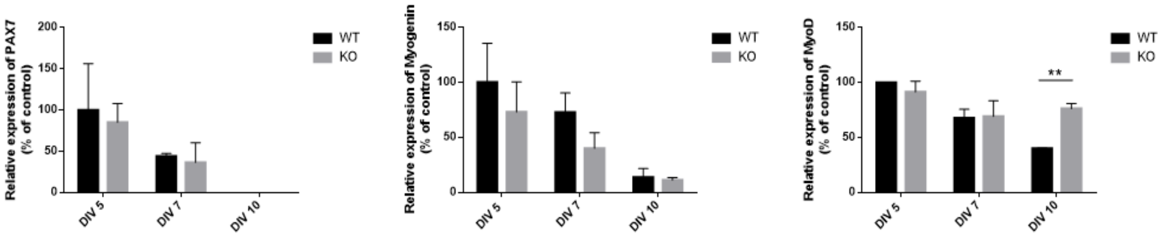

Figure S2. Densitometric quantification of proteins presented in Figure 1D. Analysis was performed using ImageJ software with respect to the amount of GAPDH. The data are expressed as mean ±SD versus control (WT cells); t-test, $^{**}p \leq 0.01$.
