# Supplementary material for: Formation of Aberrant Myotubes by Myoblasts Lacking Myosin VI Is Associated with Alterations in the Cytoskeleton Organization, Myoblast Adhesion and Fusion
Source: Cells. 2020 Jul 11;9(7):1673. doi: 10.3390/cells9071673 (PMC7408620; doi:10.3390/cells9071673)
Supplement: Supplementary file 1 [file cells-09-01673-s001.zip › Supplementary Figure 3.pdf]

Lehka et al. Supplementary Figure 3

Figure S3. Analysis of cytoskeletal proteins.

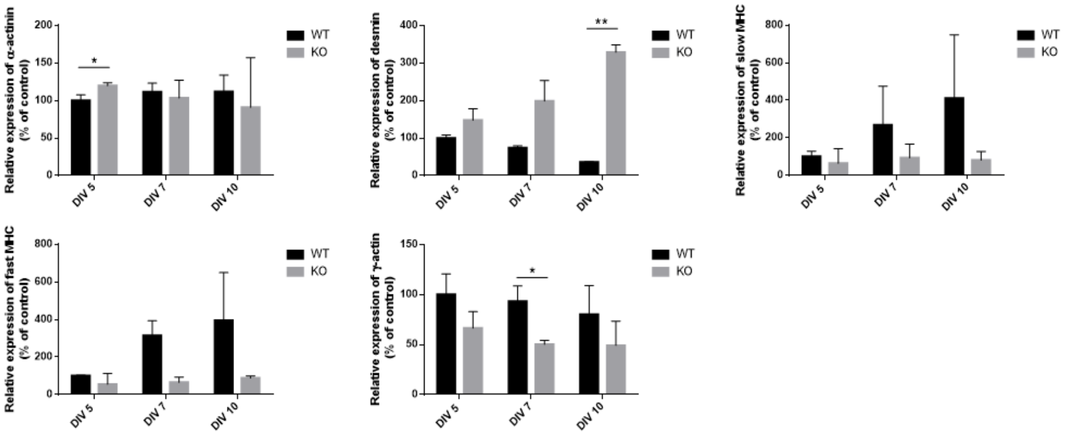

Figure S3A. Densitometric quantification of proteins presented in Figure 2A. Analysis was performed using ImageJ software with respect to the amount of GAPDH. The data are expressed as mean ±SD versus control (WT cells); t-test, **p*≤0.05, ***p*≤0.01.

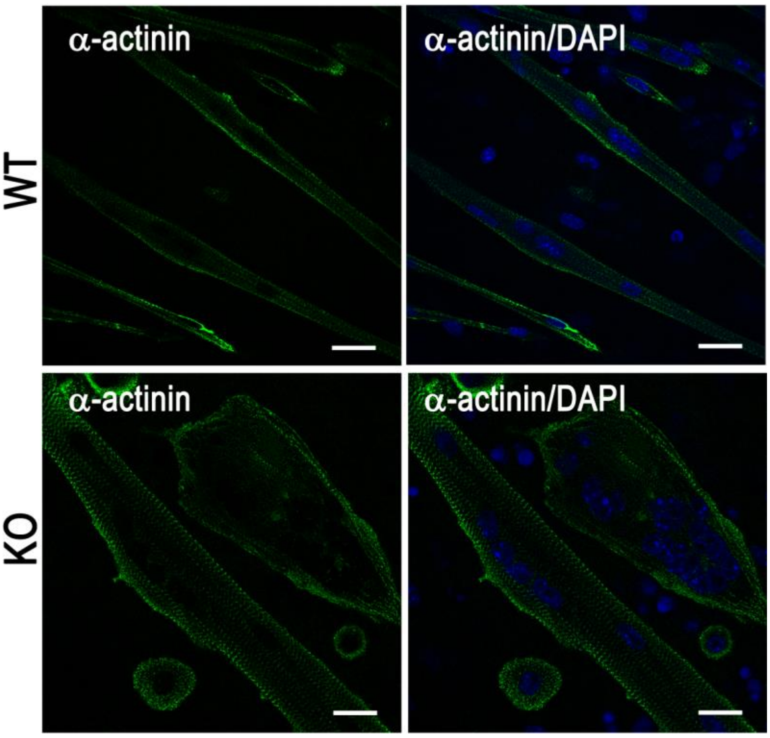

Figure S3B. Primary mouse myoblasts cultured for 10 days. Myoblasts derived from hindlimb muscles of 3-month old heterozygous (WT) and *Snell's waltzer* (KO) mice were stained for α-actinin (green) and nuclei (blue) as described in the Materials and Methods section. These are the 0.35 µm thick confocal images of both types cell centers. Bars, 10 µm.
