# Supplementary material for: Formation of Aberrant Myotubes by Myoblasts Lacking Myosin VI Is Associated with Alterations in the Cytoskeleton Organization, Myoblast Adhesion and Fusion
Source: Cells. 2020 Jul 11;9(7):1673. doi: 10.3390/cells9071673 (PMC7408620; doi:10.3390/cells9071673)
Supplement: Supplementary file 1 [file cells-09-01673-s001.zip › Supplementary Figure 5.pdf]

Lehka et al. Supplementary Figure 5

Figure S5. Densitometric analysis of the levels of proteins involved in myoblast membrane fusion and repair.

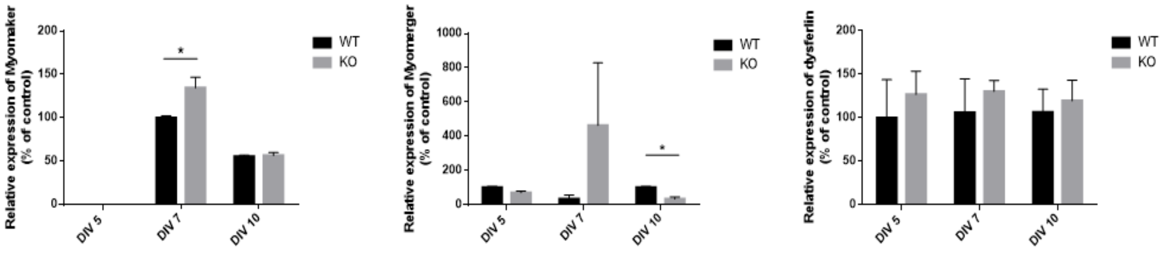

Figure S5. Densitometric quantification of proteins presented in Figure 4A. Analysis was performed using ImageJ software with respect to the amount of GAPDH. The data are expressed as mean ±SD versus control (WT cells); t-test, *$p \leq 0.05$.
